# The selective electrochemical fluorination of *S*-alkyl benzothioate and its derivatives

**DOI:** 10.3762/bjoc.14.27

**Published:** 2018-02-12

**Authors:** Shunsuke Kuribayashi, Tomoyuki Kurioka, Shinsuke Inagi, Ho-Jung Lu, Biing-Jiun Uang, Toshio Fuchigami

**Affiliations:** 1 Department of Electronic Chemistry, Tokyo Institute of Technology, 4259 Nagatsuta-cho, Midori-ku, Yokohama 226-8502, Japan; 2 Department of Chemical Science and Engineering, School of Materials and Chemical Technology, Tokyo Institute of Technology, 4259 Nagatsuta-cho, Midori-ku, Yokohama 226-8502, Japan; 3 Department of Chemistry, National Tsing Hua University, 101, Sec 2, Kuang Fu Rd., Hsinchu 300, Taiwan, R.O.C

**Keywords:** anodic cyclization, diastereoselective fluorination, electrosynthesis, fluorobenzothiophenone, selective fluorination

## Abstract

We herein report that the regioselective anodic fluorination of *S*-alkyl benzothioate and its derivatives in various aprotic solvents using Et_3_N·*n*HF (*n* = 3–5) and Et_4_NF·*n*HF (*n* = 3–5) as supporting electrolyte and a fluorine source successfully provided the corresponding α-fluorinated products in moderate yields. Dichloromethane containing Et_4_NF·4HF was found to be the most suitable combination as electrolytic solvent and supporting salt as well as fluorine source for the anodic fluorination. The electrochemical fluorination of cyclic benzothioates such as benzothiophenone was also achieved.

## Introduction

Due to the interesting properties of fluorine atoms and carbon–fluorine bonds, organofluorine compounds are widely used in various fields like pharmaceutical chemistry, agrochemistry, and materials sciences [1–2]. Therefore, the selective fluorination of organic compounds is highly useful for the development of novel organofluorine compounds. Although a number of fluorination reagents have been developed so far, they have still some problems, i.e., they are costly, difficult to handle, and explosive [3–4]. On the other hand, Rozhkov and Laurent reported an electrochemical partial fluorination of naphthalene and olefins about 30 years ago [5–6]. However, at that time, there has been no report on the anodic fluorination of heteroatom-containing compounds. At almost the same time, we found that the anodic α-methoxylation of organosulfur and amino compounds was generally enhanced by the presence of an α-electron-withdrawing group (EWG) such as a CF_3_ group. Here, the deprotonation of an anodically generated radical cation intermediate is accelerated by an EWG [7–8]. Based on these facts, we successfully achieved the first anodic fluorination of sulfides having various EWGs at their α-position using Et_3_N·3HF [9–10]. Since then, we have systematically studied the electrochemical fluorination of numerous organic compounds, heterocycles, and macromolecules by using various fluoride salts such as Et_3_N·*n*HF (*n* = 3–5) and Et_4_NF·*n*HF (*n* = 3–5) [11–19].

On the other hand, Simonet and co-workers reported the anodic fluorination of alkyl phenyl sulfides having an EWG on the phenyl group in Et_3_N·3HF/MeCN to provide α-monofluorinated products in moderate yields [20]. We also achieved the anodic fluorination of benzyl and ethyl thiocyanates as well as *O*-methyl *S*-aralkyl thiocarbonates by using the anodically stable Et_3_N·5HF and Et_4_NF·4HF [21–22]. In both cases, an EWG attached to the phenyl group and the electron-withdrawing SCN group both contribute to the generation of the cation resulting in a regioselective α-fluorination. Based on these findings, we anticipated that the α-cationic intermediate could also be generated anodically from *S*-alkyl benzothioates. Moreover, we previously successfully carried out an anodic fluorodesulfurization of *S*-aryl thiobenzoates, and found that the indirect electrolysis using a triarylamine mediator gave much better yields of benzoyl fluorides compared to the direct electrolysis [23]. Therefore, we became interested in the anodic behavior of *S*-alkyl benzothioates in the presence of fluoride ions. With this in mind and in order to provide an additional application of our electrochemical fluorination, we have studied the anodic fluorination of *S*-alkyl benzothioate and its derivatives as well as its cyclic analogues such as benzothiophenone.

## Results and Discussion

### Oxidation potentials of *S*-butyl benzothioates

**Figure 1:**
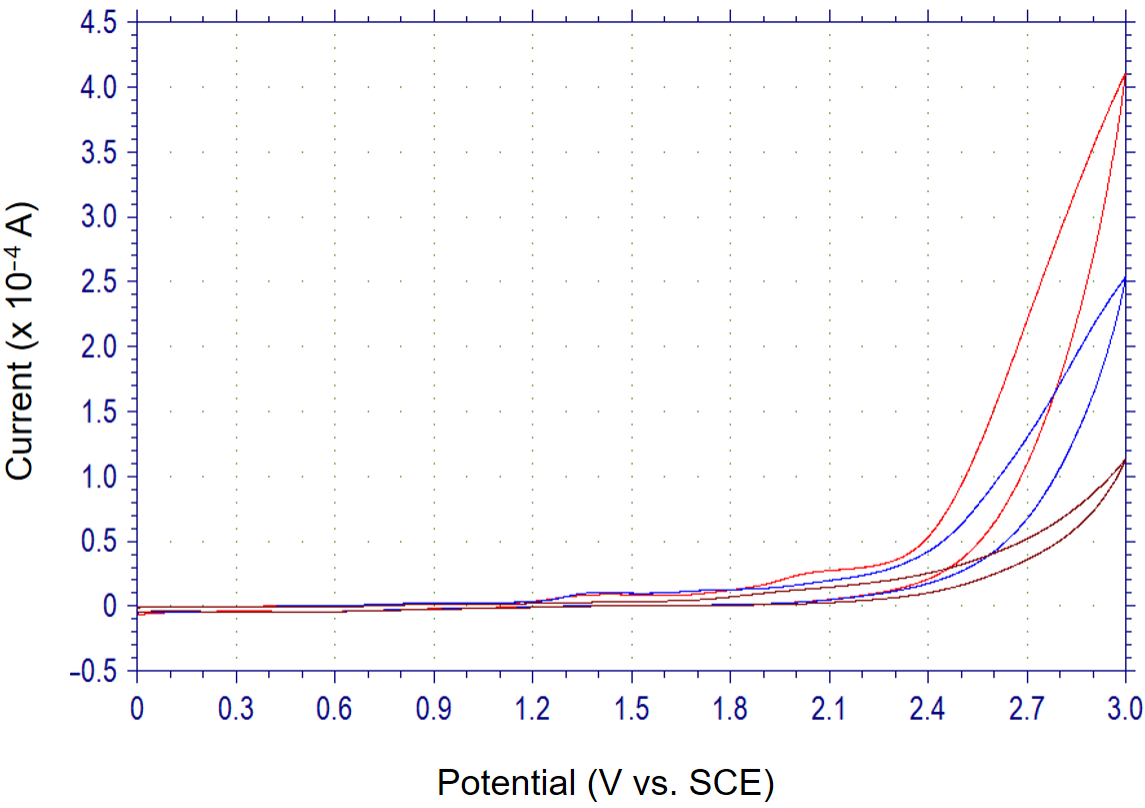
Cyclic voltammograms of 0.1 M Bu_4_NBF_4_/MeCN with a Pt disk working electrode in the absence (brown line) and presence of 5.0 mM (blue line) and 10.0 mM **1a** (red line). Scan rate of 100 mV/s.

**Figure 2:**
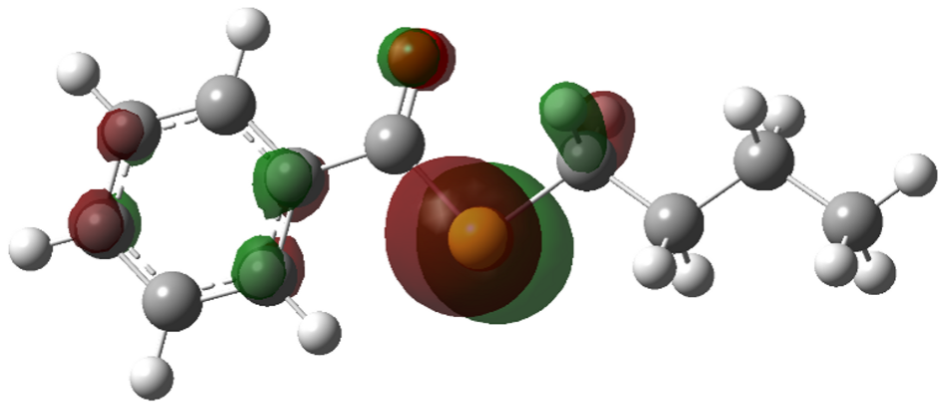
Calculated HOMO diagram of **1a**.

At first, the anodic oxidation potentials of *S*-butyl benzothioate (**1a**), *S*-butyl *p*-chlorobenzothioate (**1b**), and *S*-butyl *p*-fluorobenzothioate (**1c**) were determinded by cyclic voltammetry (CV). The compounds did not exhibit clear oxidation peaks, however, discharge started at around +2.3 V to +2.4 V vs SCE as exemplified in Figure 1 (CV curves of **1a**). Thus, it was found that these compounds have rather high oxidation potentials. DFT calculation of **1a** indicated that the highest occupied molecular orbital (HOMO) is located at the sulfur atom (Figure 2). Although sulfur atoms are easily oxidized, the oxidation potential of **1a** was found to be extremely high, that is due to the strongly electron-withdrawing benzoyl group attached to the sulfur atom. Benzyl thiocyanate is also known to be oxidized at a high potential which is similar to that of **1a** [21].

### Anodic fluorination of *S*-butyl benzothioates

Next, we carried out the anodic fluorination of **1a** as a model compound under various electrolytic conditions. Electrolysis was performed at a constant current (40 mA) with platinum electrodes (2 cm × 2 cm) in several solvents containing various fluoride salts in an undivided cell at room temperature and current was passed basically until **1a** was consumed. The results are summarized in Table 1.

**Table 1:** Electrochemical fluorination of *S*-butyl benzothioate derivatives.

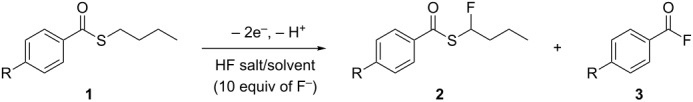							

entry	compound	R	supporting electrolyte	solvent	electricity (F/mol)	yield (%)^a^	
						**2**	**3**

1	**1a**	H	Et_3_N·3HF	CH_2_Cl_2_	4.5	55	−
2	**1a**	H	Et_3_N·3HF	CH_3_CN	7.0	26	−
3	**1a**	H	Et_3_N·3HF	DME	2.0	0^b^	−
4	**1a**	H	Et_3_N·3HF	CH_3_NO_2_	7.0	29	−
5	**1a**	H	Et_3_N·4HF	CH_2_Cl_2_	4.5	56	−
6	**1a**	H	Et_3_N·5HF	CH_2_Cl_2_	4.0	43	−
7	**1a**	H	Et_4_NF·3HF	CH_2_Cl_2_	5.4	57	−
8	**1a**	H	Et_4_NF·4HF	CH_2_Cl_2_	4.0	55	−
9	**1a**	H	Et_4_NF·5HF	CH_2_Cl_2_	4.0	38	−
10^c^	**1a**	H	Et_4_NF·4HF	CH_2_Cl_2_	4.0	67 (61)	12
11^c^	**1b**	Cl	Et_4_NF·4HF	CH_2_Cl_2_	6.0	51 (45)	20
12^c^	**1c**	F	Et_4_NF·4HF	CH_2_Cl_2_	4.0	60 (50)	9

^a^Determined by ^19^F NMR. Isolated yield is shown in parentheses. ^b^Substrate **1a** was mostly recovered. ^c^Twenty equiv of the fluoride source were used.

Regardless of the electrolytic conditions, the anodic fluorination proceeded to provide the desired fluorinated product **2a** except for the electrolysis performed in dimethoxyethane (DME) as the solvent (Table 1, entry 3). The oxidation potential of **1a** is rather high, while that of DME is relatively low [24]. Therefore, DME seems to be oxidized prior to **1a** resulting in no formation of **2a**, and **1a** was mostly recovered even when passing the theoretical amount of electricity (2 F/mol). Among the solvents tested, CH_2_Cl_2_ was found to be the best and the desired fluorinated product **2a** was obtained in 55% yield (Table 1, entry 1). In this case, the starting **1a** was consumed completely at 4.5 F/mol. It is well known that the fluoride salt, Et_3_N·3HF is easily oxidized (2.0 V vs Ag/Ag^+^) because it contains a considerable amount of free Et_3_N [25]. However, the fluorination proceeded well particularly in a CH_2_Cl_2_ solution containing Et_3_N·3HF. In contrast, when MeCN and MeNO_2_ were used as an electrolytic solvent, the yield was decreased considerably (Table 1, entries 2 and 4). This is because of a low conversion of **1a** in these solvents and indeed a large amount of **1a** was recovered.

Subsequently the effect of different fluoride salts on the fluorination was investigated similarly (Table 1, entries 5–9). Regardless of the fluoride salts, product **2a** was obtained in moderate yield, however, using Et_4_NF·4HF gave the best current efficiency for the formation of **2a** (Table 1, entry 8). Increasing the amount of Et_4_NF·4HF from 10 to 20 equiv, the product yield was also increased from 55% to 67% (Table 1, entry 10). However, in this case, C–S bond cleavage also took place to form benzoyl fluoride (**3a**) as a byproduct in considerable amounts. In all cases, no fluorination at the phenyl group took place.

Then, this anodic fluorination was extended to *p*-chloro- and *p*-fluorobenzothioates, (**1b**) and (**1c**), using 20 equiv of Et_4_NF·4HF as the supporting electrolyte (Table 1, entries 11 and 12). In both cases, a fluorine atom was introduced selectively to the α-position to provide **2b** and **2c** in moderate yields. However, the corresponding benzoyl fluorides **3b** and **3c** were also formed similarly to the case of **1a**.

### Anodic fluorination of *S*-(ω-substituted alkyl) benzylthioates

Initially, the oxidation potentials of various *S-*(substituted alkyl) benzylthioates **1d**–**j** were determined by CVs in 0.1 M Bu_4_NBF_4_/MeCN similarly to the case of **1a** and the results are collected in Table 2. Unexpectedly, no relationship between the substituents and the oxidation potentials could be observed. Next, the anodic fluorination of **1d**–**j** was carried out at a constant current in Et_4_NF·4HF/CH_2_Cl_2_ and the results are summarized also in Table 2.

**Table 2:** Oxidation potentials and electrochemical fluorination of *S*-substituted alkyl benzothioates.

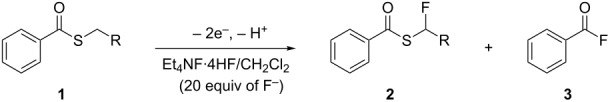						

entry	compound	R	oxidation potential (V vs SCE)	electricity (F/mol)	yield (%)^a^	
					**2**	**3**

1	**1a**	*n*-Pr-	2.3	4.0	67 (61)	12
2	**1d**	NCCH_2_CH_2_-	2.1	6.0	43 (35)	12
3	**1e**	MeOCH_2_CH_2_-	2.0	6.0	33 (29)	19
4	**1f**	EtOOC-	2.2	6.0	36 (29)	12
5	**1g**	HC≡C-	2.3	8.0	40 (37)	10
6	**1h**	Ph-	2.1	4.0	56 (46)	5
7	**1i**	PhCH_2_-	2.2	6.0	10 (7)	5
8	**1j**	*p*-CF_3_C_6_H_4_CH_2_-	2.1	6.0	48 (40)	12

^a^Determined by ^19^F NMR. Isolated yields are shown in parentheses.

**Figure 3:**
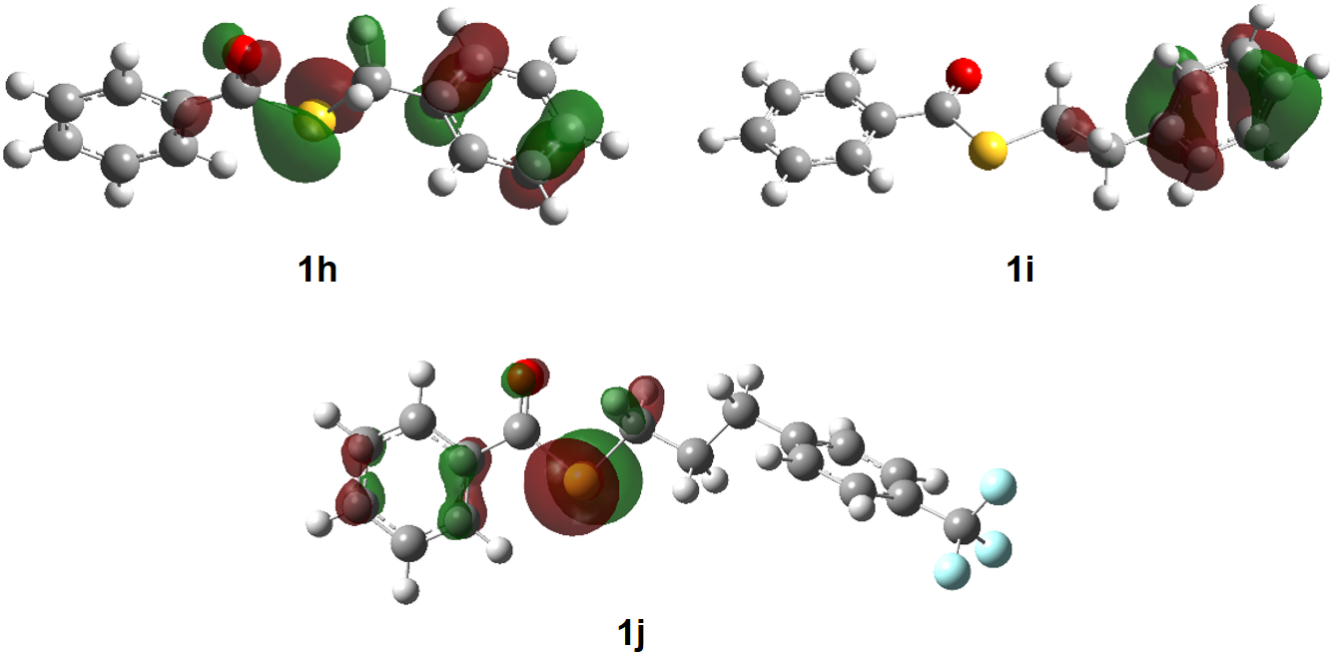
Calculated HOMO diagrams of **1h**, **1i** and **1j**.

**Scheme 1:**
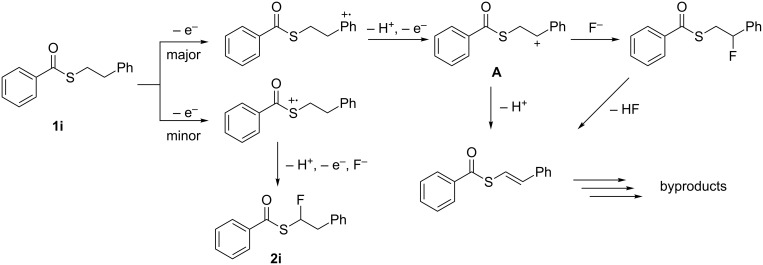
Plausible reaction paths of the anodic oxidation of **1i** in Et_4_NF·4HF/CH_2_Cl_2_.

Regardless of the substituents, the anodic fluorination took place to afford the corresponding α-fluorinated products **2** in moderate to reasonable yields along with benzoyl fluoride as byproduct (Table 2). Generally, no fluorination of the phenyl ring was observed. In the case of **1h**, electron transfer seems to take place from both the sulfur atom and the α-phenyl group, which was suggested by DFT calculation (Figure 3). However, regardless of the discharging sites, the same cationic intermediate is generated through two-electron oxidation and deprotonation (in α position to both the sulfur atom and phenyl group), which forms the α-fluorinated product **2h** selectively. In contrast, the electron transfer of **1i** seems to take place mainly from the β-phenyl group as indicated by DFT calculation (Figure 3). As shown in Scheme 1, an electron transfer from the β-phenyl group in **1i** followed by deprotonation and an additional electron transfer generates the benzylic cationic intermediate **A**, which affords the benzylic fluorinated product. It is known that benzylic fluorinated compounds are known to be generally prone to lose a fluoride anion [26]. On the other hand, intermediate **A** may undergo also elimination of a β-proton due to the electron-withdrawing benzothioate group prior to the reaction with fluoride as reported before [27]. As a consequence the expected α-fluorinated product **2i** was formed in an only low yield of 10%.

In order to suppress the discharge from the β-phenyl group of **1i**, a strongly electron-withdrawing CF_3_ group was introduced in the *para-*position of the phenyl group. The HOMO of **1j** was found to be located mainly at the sulfur atom as determined by DFT calculation (Figure 3). As expected, the anodic fluorination proceeded to provide the α-fluorinated product **2j** in a moderate yield of 48%.

**Scheme 2:**
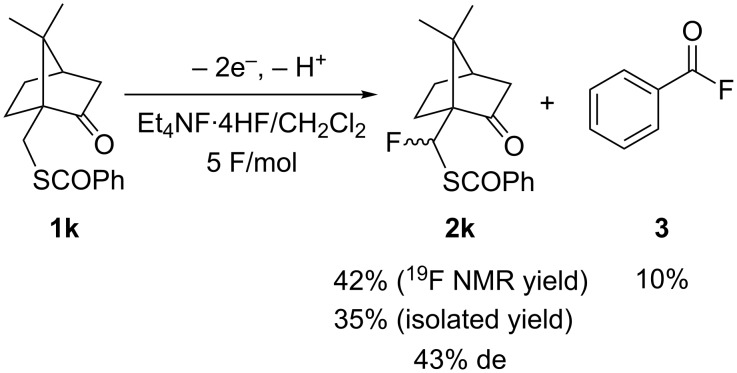
Anodic fluorination of **1k**.

Next, the anodic fluorination was performed with the benzothioate derivative **1k** having a camphor group as a chiral auxiliary. The anodic fluorination proceeded to afford the corresponding α-fluoro products as a diastereoisomeric mixture (43% de) in a reasonable yield 42% along with benzoyl fluoride as byproduct (Scheme 2).

**Scheme 3:**
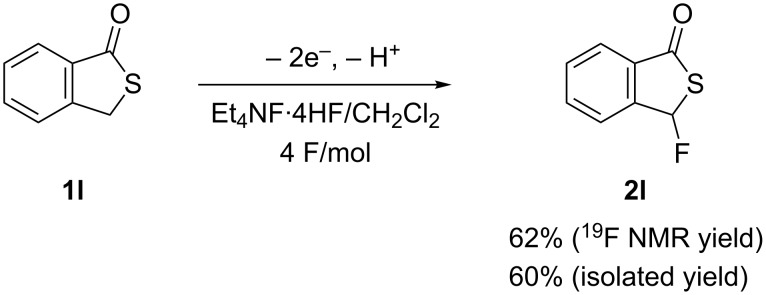
Anodic fluorination of cyclic derivative **1l**.

We next also carried out the anodic fluorination of a cyclic benzothioate namely benzothiophenone **1l**. In this case, the fluorination took place predominantly at the benzylic position to afford the fluorinated product **2l** in 60% isolated yield (Scheme 3). With this substrate, neither C–S bond cleavage nor benzene ring fluorination took place at all.

**Scheme 4:**
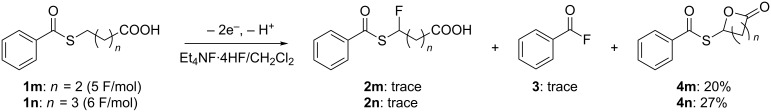
Anodic oxidation of **1m** and **1n** in Et_4_NF·4HF/CH_2_Cl_2_.

Finally, the anodic fluorination of benzothioates having a γ and δ-carboxyl group, **1m** and **1n**, was examined. The anodic oxidation of **1m** and **1n** proceeded; however, α-fluorination did not occur. In both cases, an anodic intramolecular cyclization took place to give the corresponding lactone derivatives in reasonable yields as shown in Scheme 4.

### Reaction mechanism

**Scheme 5:**
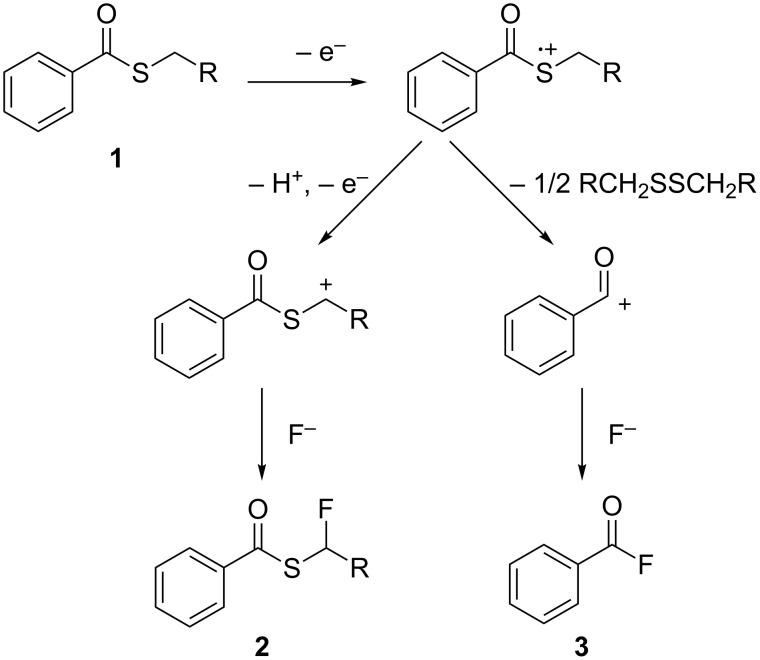
General reaction mechanism for the anodic fluorination of **1**.

In the cases of open-chain benzothioate derivatives, the anodic fluorination is initiated by an electron transfer occurring mainly from the sulfur atom of the benzothioates following two pathways (Scheme 5). The main pathway comprises the elimination of an α-proton, which is facilitated by the presence of an electron-withdrawing benzoyl group followed by another one-electron oxidation to generate a cationic intermediate which upon reaction with fluoride forms the α-fluorinated products. On the other hand, a minor pathway involves a C–S bond cleavage to form benzoyl fluoride as is observed in the case of the anodic oxidation of *S*-aryl benzothioates [23].

**Scheme 6:**
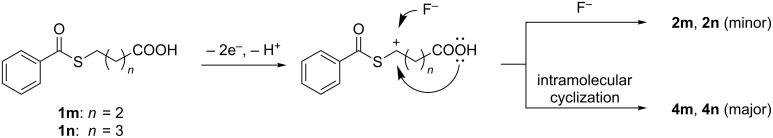
Reaction mechanism for the anodic oxidation of carboxylic acids **1m** and **1n** in the presence of a fluoride source.

In the case of *S*-alkyl benzothioates bearing a carboxyl group at the γ and δ-position with respect to the sulfur atom, after generation of an α-cationic intermediate, the intramolecular cyclization seems to take place faster than combination with a fluoride ion as shown in Scheme 6. In support of this, we have reported a fluoride ion-promoted anodic cyclization of α-(phenylthio)-*N*-phenyl- and α-(phenylthio)-*N*-benzylacetamides [28] as well as 2-(*tert*-butoxycarbonyl)oxy-3,3,3-trifluoropropyl phenyl sulfide [29].

## Conclusion

In summary, we have achieved the regioselective anodic fluorination of various *S*-alkyl and *S*-substituted alkyl benzothioate derivatives in Et_4_NF·4HF/CH_2_Cl_2_ and a fluorine atom was selectively introduced in the α-position to the sulfur atom. Under the conditions, a camphor-substituted analogue was anodically fluorinated with moderate diastereoselectivity. Moreover, the anodic fluorination of a cyclic benzothioate such as benzothiophenone was also successfully demonstrated. In contrast, the anodic fluorination of *S*-(ω-carboxy)alkyl benzothioates afforded intramolecular cyclization products like lactones instead of the corresponding α-fluorinated products.

## Experimental

### General information

^1^H, ^13^C and ^19^F NMR spectra were recorded on a JEOL JNM EX-270 (^1^H: 270 MHz, ^13^C: 67.8 MHz, ^19^F: 254.05 MHz) spectrometer in CDCl_3_. The chemical shifts for ^1^H, ^13^C and ^19^F NMR spectra are given in δ (ppm) from internal TMS, CDCl_3_ and monofluorobenzene, respectively. Cyclic voltammetry was performed using an ALS Instrument model 600A. Preparative electrolysis experiments were carried out with Metronnix Corp. (Tokyo) constant current power supply model 5944 by monitoring electricity with a Hokutodenko Coulomb/Ampere-hour meter HF-201.

### Cyclic voltammetry measurements

Cyclic voltammetry was carried out in 0.1 M Bu_4_NBF_4_/MeCN using a glass cell. A platinum disk electrode (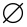 = 0.8 mm) was used as a working electrode. A platinum plate (1 cm × 1 cm) was used as a counter electrode and a saturated calomel electrode was used as a reference electrode. Electrolyte solutions for cyclic voltammetry were deoxygenated with bubbling N_2_ gas before use.

### Materials

The starting materials were prepared according to the literature procedures [21,23,30–31]. The known compounds, **1a**,**1b** [32], **1c** [33], **1f–1h** [34], **1i** [35], **1k** [36], **1l** [31], **1m** [37] and **1n** [38] were characterized by comparison of the spectral data with those reported in the literature.

### General procedure for the anodic fluorination

Analogous as described in [17], the anodic oxidation of a substrate (1 mmol) was carried out in a plastic undivided cell equipped with platinum anode and cathode (2 cm × 2 cm) containing 10 mL of HF salt (20 equiv of F^−^ to substrate/solvent) at room temperature. A constant current (40 mA) was passed until the starting material was mostly consumed (monitored by TLC). After electrolysis, the electrolytic solution was passed through a short column filled with silica gel using EtOAc as an eluent to remove the HF salt. The fluorinated product was further purified by silica gel column or preparative thin layer chromatography using a solution of hexane/EtOAc (20:1 to 1:1) as an eluent. The yield of the fluorinated products were estimated by ^19^F NMR using monofluorobenzene as an internal standard. The known fluorinated products, benzoyl fluoride [39], *p*-chlorobenzoyl fluoride [40] and *p*-fluorobenzoyl fluoride [41] were identified by comparison with ^19^F NMR and MS spectral data of the authentic samples.

## Supporting Information

File 1 Characterization data.
