# Supplementary material for: The selective electrochemical fluorination of S-alkyl benzothioate and its derivatives
Source: Beilstein J Org Chem. 2018 Feb 12;14:389–96. doi: 10.3762/bjoc.14.27 (PMC5827776; doi:10.3762/bjoc.14.27)
Supplement: File 1 — Characterization data. [file Beilstein_J_Org_Chem-14-389-s001.pdf]

Supporting Information

for

The selective electrochemical fluorination of *S*-alkyl benzothioate and its derivatives

Shunsuke Kuribayashi[1], Tomoyuki Kurioka[2], Shinsuke Inagi[2], Ho-Jung Lu[3], Biing-Jiun Uang[3], and Toshio Fuchigami*[1]

Address: [1]Department of Electronic Chemistry, Tokyo Institute of Technology, 4259 Nagatsuta-cho, Midori-ku, Yokohama 226-8502, Japan, [2]Department of Chemical Science and Engineering, School of Materials and Chemical Technology, Tokyo Institute of Technology, 4259 Nagatsuta-cho, Midori-ku, Yokohama 226-8502, Japan and [3]Department of Chemistry, National Tsing Hua University, 101, Sec 2, Kuang Fu Rd., Hsinchu 300, Taiwan, R.O.C.

Email: Toshio Fuchigami - fuchi@echem.titech.ac.jp

*Corresponding author

Characterization data

S-(3-Cyanopropyl) benzothioate (**1d**)

^{1}H NMR (270 MHz, $CDCl_3$): δ = 7.97-7.94 (m, 2H), 7.62-7.56 (m, 1H), 7.49-7.27 (m, 2H), 3.19 (t, *J* = 7.0 Hz, 2H), 2.50 (t, *J* = 7.0 Hz, 2H), 2.06 (m, 2H). ^{13}C NMR(68 MHz, $CDCl_3$): δ = 190.99, 136.46, 133.60, 128.60, 127.14, 118.84, 27.35, 25.62, 16.11. HRMS(EI): *m/z* [M^+] calcd for $C_{11}H_{11}NOS$: 205.0561; Found: 205.0561.

S-(3-Methoxypropyl) benzothioate (**1e**)

^{1}H NMR (270 MHz, $CDCl_3$): δ = 7.98-7.95 (m, 2H), 7.59-7.53 (m, 1H), 7.47-7.41 (m, 2H), 3.49 (t, *J* = 6.2 Hz, 2H), 3.35 (s, 3H), 3.15 (t, *J* = 7.3 Hz, 2H), 2.00-1.90 (m, 2H). ^{13}C NMR (68 MHz, $CDCl_3$): δ = 191.59, 136.89, 133.09, 128.37, 126.96, 70.83, 58.41, 29.45, 25.64. HRMS(EI): *m/z* [M^+] calcd for $C_{11}H_{14}O_2S$: 210.0715; Found: 210.0710.

S-4-(Trifluoromethyl)phenethyl benzothioate (**1j**)

^{1}H NMR (270 MHz, $CDCl_3$): δ = 7.97-7.94 (m, 2H), 7.61-7.34 (m, 7H), 3.32 (t, *J* = 7.6 Hz, 2H), 3.03 (t, *J* = 7.0 Hz, 2H). ^{13}C NMR (68 MHz, $CDCl_3$): δ = 191.58, 143.99, 136.90, 133.50, 128.99, 128.94, 128.65, 128.36, 127.20, 125.46 (q, *J* = 3.86 Hz). ^{19}F NMR (254 MHz, $CDCl_3$): δ = 14.06 (s). HRMS(EI): *m/z* [M^+] calcd for $C_{16}H_{13}F_3OS$: 310.0639; Found: 310.0634.

S-(1-Fluorobutyl) benzothioate (**2a**)

^{1}H NMR (270 MHz, $CDCl_3$): δ = 7.96-7.93 (m, 2H), 7.61-7.56 (m, 1H), 7.48-7.42 (m, 2H), 6.64-6.40 (m, 1H), 2.14-1.94 (m, 2H), 1.64-1.50 (m, 2H), 1.00 (t, *J* = 7.56 Hz, 3H). ^{13}C NMR (68 MHz, $CDCl_3$): δ = 188.75, 136.23, 133.93, 128.69, 127.49, 95.24 (d, *J* = 215.80 Hz), 36.62 (d, *J* = 21.76 Hz), 18.64 (d, *J* = 3.39 Hz), 13.46. ^{19}F NMR (254 MHz, $CDCl_3$): δ = -76.99--77.33 (m). HRMS(EI): *m/z* [M^+] calcd for $C_{11}H_{13}FOS$: 212.0671; Found: 212.0672.

S-(1-Fluorobutyl) 4-chlorobenzothioate (**2b**)

^{1}H NMR (270 MHz, $CDCl_3$): δ = 7.87 (d, *J* = 6.75 Hz, 2H), 7.43 (d, *J* = 6.75 Hz, 2H), 6.62-6.38 (m, 1H), 2.14-1.92 (m, 2H), 1.64-1.50 (m, 2H), 0.98 (t, *J* = 4.46 Hz, 3H). ^{13}C NMR (68 MHz, $CDCl_3$): δ = 187.63, 140.38, 134.53, 129.02, 128.82, 95.25 (d, *J* = 216.28 Hz), 36.55 (d, *J* = 21.70 Hz), 18.62 (d, *J* = 3.93 Hz), 13.45. ^{19}F NMR (254 MHz, $CDCl_3$): δ = -76.93--77.28 (m). HRMS(EI): *m/z* [M^+] calcd for $C_{11}H_{12}ClFOS$: 246.0281; Found: 246.0280.

S-(1-Fluorobutyl) 4-fluorobenzothioate (**2c**)

^{1}H NMR (270 MHz, $CDCl_3$): δ = 8.01-7.96 (m, 2H), 7.19-7.10 (m, 2H), 6.62-6.39 (m, 1H), 2.15-1.89 (m, 2H), 1.64-1.50 (m, 2H), 1.01 (t, *J* = 7.43 Hz, 3H). ^{13}C NMR (68 MHz, $CDCl_3$): δ = 187.25, 166.16 (d, *J* = 255.88 Hz), 132.58 (d, *J* = 2.78 Hz), 130.13 (d, *J* = 9.49 Hz), 115.90 (d, *J* = 21.70 Hz), 95.29 (d, *J* = 216.35 Hz), 36.56 (d, *J* = 21.76 Hz), 18.63 (d, *J* = 3.39 Hz), 13.45. ^{19}F NMR (254 MHz, $CDCl_3$): δ = -46.13--46.24 (m), -86.43--86.78(m). HRMS(EI): *m/z* [M^+] calcd for $C_{11}H_{12}F_2OS$: 230.0577; Found: 230.0580.

S-(3-Cyano-1-fluoropropyl) benzothioate (**2d**)

^{1}H NMR (270 MHz, $CDCl_3$): δ = 7.95-7.92 (m, 2H), 7.66-7.61 (m, 1H), 7.52-7.46 (m, 2H), 6.70-6.47 (m, 1H), 2.67-2.61 (m, 2H), 2.54-2.38 (m, 2H). ^{13}C NMR (68 MHz, $CDCl_3$): δ = 187.29, 135.60, 134.43, 128.88, 127.63, 118.02, 93.31 (d, *J* = 216.35 Hz), 30.85 (d, *J* = 23.39 Hz), 13.62 (d, *J* = 4.47 Hz). ^{19}F NMR (254 MHz, $CDCl_3$): δ = -81.39--81.73 (m). HRMS(EI): *m/z* [M^+] calcd for $C_{11}H_{10}FNOS$: 223.0467; Found: 223.0468.

S-(1-Fluoro-3-methoxypropyl) benzothioate (**2e**)

^{1}H NMR (270 MHz, $CDCl_3$): δ = 7.98-7.95 (m, 2H), 7.64-7.59 (m, 1H), 7.51-7.45 (m, 2H), 6.78-6.54 (m, 1H), 3.62-3.52 (m, 2H), 3.38 (s, 3H), 2.42-2.23 (m, 2H). ^{13}C NMR (68 MHz, $CDCl_3$):

δ = 188.57, 136.16, 130.60, 130.33, 130.00, 128.79, 127.60, 91.74 (d, *J* = 208.55 Hz), 67.80, 58.81 (d, *J* = 135.60 Hz), 35.36 (d, *J* = 37.36 Hz). ^{19}F NMR (254 MHz, $CDCl_3$): δ = -79.50--79.85 (m). HRMS(EI): *m/z* [M^+] calcd for $C_{11}H_{13}FO_2S$: 228.0620; Found: 228.0619.

Ethyl 2-(benzoylthio)-2-fluoroacetate (**2f**)

^{1}H NMR (270 MHz, $CDCl_3$): δ = 7.98-7.95 (m, 2H), 7.64-7.59 (m, 1H), 7.51-7.45 (m, 2H), 6.79 (d, *J* = 49.68 Hz, 1H), 4.35 (q, *J* = 7.02 Hz, 2H), 1.35 (t, *J* = 7.20 Hz, 3H). ^{13}C NMR (68 MHz, $CDCl_3$): δ = 186.40, 166.46, 135.18, 134.65, 128.98, 127.80, 88.41 (d, *J* = 229.164 Hz), 62.83, 14.06. ^{19}F NMR (254 MHz, $CDCl_3$): δ = -89.51 (d, *J* = 49.78 Hz). HRMS(EI): *m/z* [M^+] calcd for $C_{11}H_{11}FO_3S$: 242.0413; Found: 242.0412.

S-(1-Fluoroprop-2-yn-1-yl) benzothioate (**2g**)

^{1}H NMR (270 MHz, $CDCl_3$): δ = 7.97-7.92 (m, 2H), 7.67-7.61 (m, 1H), 7.52-7.46 (m, 2H), 6.99 (dd, *J* = 50.76, 2.16 Hz, 1H), 3.02 (dd, *J* = 4.86, 2.16 Hz, 1H). ^{13}C NMR (68 MHz, $CDCl_3$): δ = 187.00, 135.20, 134.58, 128.97, 127.70, 83.16, 77.01 (t, *J* = 31.80 Hz), 75.91 (d, *J* = 29.56 Hz). ^{19}F NMR (254 MHz, $CDCl_3$): δ = -74.99 (dd, *J* = 50.8, 4.60 Hz). HRMS(EI): *m/z* [M^+] calcd for $C_{10}H_7FOS$: 194.0202; Found: 194.0196.

S-(α-Fluorobenzyl) benzothioate (**2h**)

^{1}H NMR (270 MHz, $CDCl_3$): δ = 7.98-7.35 (m), 6.00 (d, *J* = 50.08 Hz, 1H). ^{13}C NMR (68 MHz, $CDCl_3$): δ = 188.38, 135.86, 133.03, 129.80, 128.74, 128.24, 127.73, 126.88, 124.58, 93.27 (d, *J* = 163.33 Hz). ^{19}F NMR (254 MHz, $CDCl_3$): δ = -73.83 (d, *J* = 50.04 Hz). HRMS(EI): *m/z* [M^+] calcd for $C_{14}H_{11}FOS$: 246.0515; Found: 246.0514.

S-(1-Fluoro-2-phenylethyl) benzothioate (**2i**)

^{1}H NMR (270 MHz, $CDCl_3$): δ = 8.00-7.97 (m, 2H), 7.62-7.56 (m, 1H), 7.49-7.36 (m, 7H), 5.59 (dt, *J* = 47.25, 4.32 Hz, 1H), 3.73-3.39 (m, 2H). ^{13}C NMR (68 MHz, $CDCl_3$): δ = 191.02, 138.36, 133.67, 128.83, 128.69, 128.61, 127.32, 125.60, 125.50, 92.77 (d, *J* = 175.06 Hz), 35.33 (d, *J* = 26.24 Hz). ^{19}F NMR (254 MHz, $CDCl_3$): δ = -97.77--98.12 (m). HRMS(EI): *m/z* [M^+] calcd for $C_{15}H_{13}FOS$: 260.0671; Found: 260.0672.

S-(1-Fluoro-2-(4-(trifluoromethyl)phenyl)ethyl) benzothioate (**2j**)

^{1}H NMR (270 MHz, $CDCl_3$): δ = 7.94-7.90 (m, 2H), 7.63-7.42 (m, 7H), 6.72 (dt, *J* = 49.68, 5.94 Hz, 1H), 3.43 (dd, *J* = 19.98, 5.94 Hz, 2H). ^{13}C NMR (68 MHz, $CDCl_3$): δ = 188.11, 139.24, 135.98, 134.24, 130.00, 129.48, 128.84, 127.65, 125.55 (q, *J* = 3.73 Hz), 94.98 (d, *J* = 219.13 Hz), 40.90 (d, *J* = 22.85 Hz). ^{19}F NMR (254 MHz, $CDCl_3$): δ = 14.06 (s, 3F), -76.81 (dt, *J* = 50.04, 20.32 Hz, 1F). HRMS(FAB): *m/z* [$M+Na^+$] calcd for $C_{16}H_{12}F_4OSNa$: 351.0443; Found: 351.0447.

S-((7,7-Dimethyl-2-oxobicyclo[2.2.1]heptan-1-yl)fluoromethyl) benzothioate (**2k**)

diastereomeric mixture (2.5:1)

^{1}H NMR (270 MHz, $CDCl_3$): δ = 8.01-7.97 (m, 2H), 7.64-7.50 (m, 1H), 7.48-7.45 (m, 2H), 6.80 (d, *J* = 48.87 Hz, 1H), 2.50 (dt, *J* = 14.58, 3.78 Hz, 1H), 2.25-2.08 (m, 3H), 1.95 (d, *J* = 18.63Hz, 1H), 1.73-1.46 (m, 2H), 1.21 (s, 3H), 1.10 (s, 3H). ^{13}C NMR (68 MHz, $CDCl_3$): δ = 213.25, 188.20, 136.23, 132.91, 129.95, 127.58, 95.06 (d, *J* = 208.49 Hz), 63.62 (d, *J* = 21.22 Hz), 48.58, 44.34 (d, *J* = 13.42 Hz), 25.78, 21.97, 21.42, 20.82, 20.19. ^{19}F NMR (254 MHz, $CDCl_3$): δ = -82.33 (d, *J* = 48.01 Hz), -85.11 (d, *J* = 49.78 Hz). HRMS(EI): *m/z* [M^+] calcd for $C_{17}H_{19}FO_2S$: 306.1090; Found: 306.1091.

3-Fluorobenzo[*c*]thiophen-1(3*H*)-one (**2l**)

^{1}H NMR (270 MHz, $CDCl_3$): δ = 7.80-7.57 (m, 4H), 7.27 (d, *J* = 58.59 Hz, 1H). ^{13}C NMR (68 MHz, $CDCl_3$): δ = 192.90 (d, *J* = 2.78 Hz), 144.48 (d, *J* = 16.75 Hz), 134.35 (d, *J* = 2.24 Hz), 131.02 (d, *J* = 2.78), 126.42 (d, *J* = 1.70 Hz), 123.75 (d, *J* = 1.15 Hz), 97.85 (d, *J* = 217.43 Hz). ^{19}F NMR (254 MHz, $CDCl_3$): δ = -76.25 (d, *J* = 58.93 Hz). HRMS(EI): *m/z* [M^+] calcd for C_8H_5FOS: 168.0045; Found: 168.0049.

S-(5-Oxotetrahydrofuran-2-yl) benzothioate (**4m**)

^{1}H NMR (270 MHz, $CDCl_3$): δ = 7.95-7.91 (m, 2H), 7.66-7.60 (m, 1H), 7.52-7.46 (m, 2H), 6.47-6.42 (m, 1H), 2.88-2.76 (m, 1H), 2.73-2.64 (m, 2H), 2.50-2.40 (m, 1H). ^{13}C NMR (68 MHz, $CDCl_3$): δ = 188.68, 175.32, 135.94, 134.26, 128.87, 127.54, 80.92, 28.57, 27.91. HRMS(EI): *m/z* [M^+] calcd for $C_{11}H_{10}O_3S$: 222.0351; Found: 222.0355.

S-(6-Oxotetrahydro-2*H*-pyran-2-yl) benzothioate (**4n**)

stereoisomer (3.2:1)

^{1}H NMR (270 MHz, $CDCl_3$): δ = 8.09-7.62 (m, 2H), 7.59-7.45 (m, 3H), 6.65-6.42 (m, 1H), 2.69-1.89 (m, 6H). ^{13}C NMR (68 MHz, $CDCl_3$): δ = 188.60, 188.22, 178.26, 169.36, 136.14, 134.16, 133.80, 130.18, 128.84, 128.49, 127.65, 127.57, 81.26, 34.12, 33.02, 29.51, 28.25, 20.54, 18.54. HRMS(EI): *m/z* [M^+] calcd for $C_{12}H_{12}O_3S$: 236.0507; Found: 236.0510.
